# Motivations, Expectations and Experiences in Being a Mental Health Helplines Volunteer

**DOI:** 10.3390/ijerph15102123

**Published:** 2018-09-27

**Authors:** Frederick Sundram, Thanikknath Corattur, Christine Dong, Kelly Zhong

**Affiliations:** 1 Department of Psychological Medicine, Faculty of Medical and Health Sciences, The University of Auckland, 1023 Auckland, New Zealand; 2 Faculty of Medical and Health Sciences, The University of Auckland, 1023 Auckland, New Zealand; thanikkcorattur@gmail.com (T.C.); Christine.Dong@mercury.co.nz (C.D.); 3 Faculty of Medical and Health Sciences, School of Population Health, The University of Auckland, 1023 Auckland, New Zealand; k.zhong@auckland.ac.nz

**Keywords:** telemental health, motivations, helpline, volunteer, volunteer retention

## Abstract

Volunteers in non-government organisations are increasingly providing mental health support due to increasing demand and in the context of overstretched publicly-funded mental health services. This descriptive, cross-sectional study explored a knowledge gap in the literature of mental health telephone counselling by examining the motivation and retention determinants of helpline volunteers. In total, 25 participants were recruited across four focus groups and five individual interviews from a non-government organisation which provides a national phone counselling service to callers in New Zealand. Interviews were electronically recorded, transcribed and thematically analysed. Volunteers were found to have a high regard for their role and enjoyed many aspects including initial training, ongoing supports (formal/informal) and nature of the phone calls. However, organisational priorities/communication, disconnectedness, technological issues, lack of recognition and lack of a sense of belonging were reasons cited for intention to leave but previous mental health experiences, autonomy/flexibility, self-discovery/skills development and being there for someone else were key factors for volunteers to start and remain in their role. Understanding these crucial factors may help modulate volunteer satisfaction and retention in mental health organisations but may also potentially be relevant to other types of volunteer organisations.

## 1. Introduction

Volunteers come from a variety of backgrounds with the potential to bring a range of skills to the voluntary sector [1]. Volunteers provide their skills to several fields including mental health whereby there has been an increase in services provided by voluntary non-government organisations, and such organisations contribute to a significant portion of service provision within many countries [2]. Additionally, funding for mental health services globally appears to be low [3,4,5,6,7] despite increasing demand which places an important focus on volunteers and the valuable input they provide though costs for running these organisations are high, including recruiting, training and loss of volunteers [2]. Furthermore, government policy and the retention of volunteers needs to be guided by the evidence in the literature [8,9].

Volunteer turnover is a frequent problem in many non-government organisations [10] and attempts have been made to understand volunteer motivations and the reasons for starting and staying in their role [11,12,13,14,15,16,17,18]. The act of volunteering is usually determined by an individual’s choice [19] and is influenced by a number of reasons, for example, as seen in the Sustained Volunteerism Model [20]. Frameworks such as this help to understand the motivations to start and remain as a volunteer while volunteer retention refers to the number of volunteers who successfully complete their commitment to the organisation as well as those who renew and continue serving the organisation [21]. Volunteer retention is an important consideration for many voluntary organisations whereby high rates of volunteer turnover can affect the range and quality of services offered [22]. In addition, the first six months of a volunteer’s experience is critical towards their choice of remaining in their role [23].

There are rewards to being a volunteer which have been conceptualised as altruism versus egoism [24,25]. The altruistic approach is selfless and related to intrinsic rewards, i.e., the act benefits others as well as providing personal satisfaction. Conversely, egoism relates to the external rewards that are associated with being a volunteer, e.g., materialistic and social motives such as social networking, skill development, career prospects and recognition.

Furthermore, functional analysis of volunteering examines the context and process of volunteering and helps to understand the underlying needs and motives of volunteers because volunteers may undertake the same tasks, but for different reasons [11,26,27] and achieve different levels of motive satisfaction [28]. The construct of role identity is another important consideration as it modulates sustained volunteering. With this theory, volunteers may, or over time, develop several identities based on social interactions and expectations from others which is then internalised by the volunteer [29]. Although the influence of others is initially important, this gradually wanes and once the role is integrated as part of their self-concept, this governs future behaviour. Expanding on this further, it is possible for a volunteer to hold a general role identity such as helping others (e.g., through blood donation or working with disabled children, palliative care and dementia support). However, a specific role identity may develop where for example, the volunteer may view themselves as a blood donor, i.e., it is who they are rather than what they do. Moreover, as volunteering often occurs within the context of organisations, the goals and values of the organisation are important because when there is alignment between the organisation and volunteer, this promotes organisational commitment and sustained volunteering. Conversely, role conflict may arise when there is a mismatch [30] or impaired performance due to role ambiguity [31].

Apart from organisation context, macro-societal attributes such as prevailing cultural norms can impact on individual volunteers’ motivations, personalities and satisfaction of their roles. For example, in a society that values collectivism rather than individualism, volunteers may prefer to be part of a wider group and prioritise the goals of the group [32]. In addition, being part of a community in collectivistic societies may contribute to volunteer satisfaction and commitment [33], whereas volunteers in individualistic societies may be motivated by and draw satisfaction from factors such as career development instead. Although what motivates volunteers in either of these societies may differ, their willingness to help others or duration of volunteering may not significantly differ [33,34].

Finkelstein was able to link various facets of functional analysis, role identity and motivational orientation and summarised a construct of intrinsic and extrinsic motivations whereby the former may represent internal motives or the activity itself (e.g., expression of altruistic values or skills/self-esteem/social connection enhancement) while the latter may be related to outcomes (e.g., career development) [35]. Further, volunteer motivations do not remain static and can potentially change over time [36,37].

Overall, volunteer retention is complex and proposed to be modulated by a variety of factors [12,13,14,17,35] which can be summarised as follows: (i) individual volunteer factors (e.g., intrinsic and extrinsic motivations for volunteering); (ii) communities or groups the volunteer may belong to; (iii) aspects of the volunteer organisation; and (iv) macro-societal variables.

While there is established literature in generic volunteers, there is however limited work examining helpline volunteers in the field of mental health. Furthermore, with regard to telephone counselling, historically, research has focused on characterising callers [38,39,40] or the needs of callers, with fewer focusing on the needs of the volunteers [41,42,43]. The current study provides an opportunity to explore the perspectives of volunteers within a mental health organisation that provides helpline services to users nationally.

### Aims

This study aimed to investigate helpline volunteers’ motivations, expectations and experiences influencing the initiation, continuation, or discontinuation of volunteering in a national mental health counselling helpline organisation in New Zealand and answer these key questions:

1) What are the key motivations to start volunteering?
2) What expectations do helpline volunteers have around initial training, ongoing support and day to day work?
3) What positive or negative experiences have they had as a helplines volunteer?
4) What factors are associated with volunteer job satisfaction and intention to stay?
5) What factors are associated with intention to leave?

## 2. Materials and Methods

This study is part of a cross-sectional investigation during October–December 2014 which used both quantitative and qualitative methodologies. An online questionnaire composed of mainly quantitative responses and limited free-text qualitative sections was utilised. Both semi-structured focus group and individual in-depth face to face interviews were then separately undertaken. This paper only reports the qualitative findings (from focus groups, in-depth interviews and qualitative portions of the online questionnaire) with the quantitative results reported separately. This qualitative approach included aspects of grounded theory and overlapping contributions from post-positivist and critical research [44,45]. Ethics approval was provided by The University of Auckland Human Participants Ethics Committee (Reference: 013079).

### 2.1. Study Setting

This study was undertaken at a national phone counselling service which provides not only a helpline service to the general population but also includes service provision for specific groups via tailored helplines, e.g., adolescents and those from different cultural backgrounds. Most telephone counsellors are volunteers who have been recruited and trained by the organisation. Following recruitment, volunteers begin a two-week new volunteers training programme focused on the development of skills for answering the helplines as well as self-management. Volunteers also avail of on the floor supervision (when available) whereby supervisors provide real-time support should volunteers require it. Additionally, there is ongoing formal support whereby volunteers meet monthly as groups and can share their helpline experiences with a trained supervisor. This includes discussing any challenging interactions, debriefing and utilising helpful strategies for the future.

At the time of the study, the organisation was undergoing national restructuring. Regional branches that were previously receiving local calls were now accepting calls from across the country to maximise cost-efficiencies. Additionally, the restructuring aimed to address technological issues, inconsistencies amongst certain branches, including the training programme and quality of services provided. The target population of the study was active helpline volunteers at all national branches of this organisation whereby the majority were long-term volunteers, whereas a smaller proportion (approximately 13%) were students who turnover regularly. The researchers in the current study were independent to the national counselling service and had experience in undertaking qualitative research and working in mental health clinical environments.

### 2.2. Recruitment

Focus group invitations were sent to all active helpline volunteers in November 2014 (total sample population was *n* = 136, ≈80% female) with the intention of having up to five focus groups for the larger helpline service which focused on English-speaking adults and five individual interviews for the smaller cultural helpline service. Individual interviews were offered in English or Mandarin. A final sample of 25 volunteers was recruited: 20 to four focus groups while five were recruited for individual interviews. Convenience sampling was undertaken, and this study was exploratory in nature. No saturation point was aimed for.

### 2.3. Interviews

The same questions (Table A1) were used for focus group and individual interviews and these were initially created by the study authors and were subsequently reviewed by professionals who work in helpline organisations. Consent forms were signed prior to all interviews which were semi-structured and electronically recorded. Each focus group contained a moderator facilitating the group and two research assistants who assisted with managing the group and electronic recordings. The focus group sessions lasted 50–70 min, whereas individual interviews lasted 15–60 min with one interviewer and one research assistant.

### 2.4. Transcriptions and Data Entry

Recordings were electronically transcribed to Microsoft Word by T.C. and K.Z. Each transcript was checked against audio recordings and minor corrections were made where necessary. All participants were anonymised during this process. One individual interview that was conducted in Mandarin was transcribed in Mandarin and translated to English by one of the authors (C.D.). As participants were anonymised, it was not possible to bring study findings back to participants for trustworthiness.

### 2.5. Data Analysis

Thematic analysis was conducted with the aid of NVivo (Version 10, 2012) (QSR International Pty Ltd., Melbourne, Australia) and the general inductive approach [46] was used to organise the dataset into multiple coded blocks. A higher level of data interpretation was then performed, and responses were assessed for what might be implied or inferred. Transcripts from focus groups, in-depth individual interviews and the qualitative portion of the online survey were checked and coded independently. The independent coding by the researchers (F.S. and T.C.) was then discussed until consensus was reached to determine the final themes. Quotations from focus groups, where relevant, are provided verbatim.

## 3. Results and Discussion

### 3.1. Characteristics of the Study Participants

A total of 25 helpline volunteers aged 25–67 years participated in the interviews (all female except two males). The mean length of volunteering with the organisation was three years (range = 1–15 years). Details of focus groups are provided (Table 1) but individual interview details are omitted to preserve anonymity.

### 3.2. Qualitative Findings

#### 3.2.1. Motivations to Start Volunteering

Volunteers wanted to give back to the wider community and sought to provide what others had or had not been able to give to them. Volunteers also mentioned intrinsic motivations such as developing new skills alongside their altruistic motivations. For example, to gain skills and work experience required for future employment, as well as gaining skills in psychology.

“*… for me it was just wanting to give back … to the community, plus also learn some skills … I had teenage daughters at the time and they had some certain things they were going through, so if I could learn more skills in order to help them as well as the community then, hey*.”

“*I want to learn more about … counselling. Because I [have] always [had] the interest in psychology and [the] counselling field*.”

A variety of reasons to start volunteering aligned with the existing literature, in particular regarding intrinsic and extrinsic motivations [35]. Factors for motivation included flexibility (with low impact on volunteers’ personal lives) and expected enjoyment of the role. Within Penner’s Model [20], the motivations to volunteer have been thoughtfully mapped out, and the current study highlights various reasons for the initiation of volunteering. It can be appreciated from the results of the current study and previous literature that a start in volunteering can be planned or spontaneous. However, in the field of mental health, significant thought was placed before a decision was made. This was not perceived to be the same for the students volunteering within the organisation in the current study as the perceived motivation among university students for volunteering was extrinsic in nature as it appeared to longer-term volunteers that the students only wished to gain relevant skills from the new training programme and participate in the minimum number of hours required for their psychology course curriculum. Although youths start and continue volunteering for a variety of reasons that span intrinsic and extrinsic reasons; youths, in general, do want to make a difference but also wish to attain self-enhancement, skills and experience for their future careers [37,47,48]. However, when volunteering is a compulsory obligation, the lack of choice has been found to reduce the young person’s sense of agency and does not facilitate positive community attitudes or social behaviours [49]. Additionally, compulsory volunteering may not incentivize learning or mastery of skills [50]. While this is not surprising, commitment to volunteering is a complex process and assessing initial motivations is also necessary alongside consideration of factors for retention of an individual [37].

Personal loss, e.g., via suicide, motivated some individuals to provide support to others similarly bereaved. For others, previous volunteer work within the family, and how their “family has always been involved in volunteering”, contributed to their identity as it was a “natural part of who they are” consistent with the literature on role identity [29].

“*As well as having a number of people in my family with mental health issues [there were] a number of suicides, and I thought maybe I could be that person that makes a difference in somebody’s life*.”

The relationship between motivation to start volunteering and previous personal loss has been understudied in the literature and our study adds to the literature that individuals with past experiences of pain wish to alleviate this for others [51].

#### 3.2.2. Positive Experiences

Volunteers appreciated the supportive network enabled by the organisation during supervision and during challenging phone calls. Volunteers that had real-time supervisor support valued the learning opportunities provided by the supervisor during and after phone calls. The high-quality of the new volunteer training programme and ongoing supervision not only contributed to role satisfaction but also development of a range of skills within and beyond their volunteer role. The volunteers appreciated that skills were not only focused on counselling, but also on self-growth and self-care. They additionally enjoyed the social support of other volunteers and making a difference, helping the caller and phone calls ending on a positive note.

“*I enjoy the time being with my peers when we have group meetings or supervisions because I view us a big family [sic], we support each other and we are working towards a goal to help the community. That’s the most encouragement and satisfaction for me, being a volunteer and continue it*.”

“*… if someone calls us that day [and] they were in [a] really bad situation … [and] at the end … if they are any better than they were before, I think that’s the part I enjoyed*.”

“*… you become more self aware and you also become more aware of other [sic] … of your impact upon other people. So in my opinion … the training at this stage, it’s a valuable set of skills that you leave with*.”

A focus on personal wellbeing reduces the likelihood of volunteers leaving the organisation [52,53] and the findings in the current study provide support that ongoing supervision and the informal network around volunteers provides volunteers with the opportunity to express their views in a supported environment.

Some expectations of volunteers prior to starting their role were met, which contributed as a positive experience towards their volunteer role. Volunteers expected to develop new skills such as counselling, as well as the organisation having the appropriate structures to support this. Additionally, developing a strong supportive social network with other volunteers to create a sense of belonging was important. The findings suggest that belonging to a group with a shared purpose [32] also leads to satisfaction of the role in the mental health arena.

“*I had expectations of being part of a community … of like-minded people that you could share stories with and also, beyond the time committed to being on the phone, you would have opportunities to talk and share …*”

“*Our supervision program is awesome. I feel well supported in all aspects of my [volunteer] work*.”

#### 3.2.3. Negative Experiences

Ambiguity of task requirements, inefficient use of time and lack of appreciation by the organisation have been reported to contribute to lower volunteer satisfaction in previous work [31,54]. In the current study, several volunteers felt they were being treated as employees rather than volunteers by the organisation and issues with formal and informal recognition also arose, e.g., not being consistently recognised for their work via certificates or not being thanked for coming into the phone counselling office despite using their free time to help the wider community.

“*I think that the organisation should appreciate the difference between an employee and a volunteer. The tone used to communicate with volunteers is often inappropriate, and one’s natural reaction may well be, to ignore at best or as many volunteers have done simply to decide that this is too hard and to vote with their feet*.”

This can be partially attributed to organisational changes and the change management process, whereby volunteers have started perceiving the organisation as becoming more of a business rather than a service to the community. In a previous study, organisational changes and recognition have been highlighted as a potential contributor that could affect volunteering [55]. Indeed, the literature on organisation change has highlighted that members of an organisation may hold cognitive models of planned changes that over time, may or not match anticipated changes [56]. In the current study, some volunteers thought they brought a set of skills to the organisation but these were not explored or utilised. Consequently, volunteers felt underappreciated and affected the volunteers’ sense of belonging within the organisation.

Organisational factors including expansion or changes in facilities can also have the potential to affect a volunteer’s sense of belonging to the organisation and has been described in the literature as a component of social identity termed place identity [57]. Volunteers may be unaware of future organisational changes or the reasons for them and involving volunteers in discussions regarding future organisational changes as well as gaining feedback from them prior to implementing these changes may be helpful as part of information sharing and co-design [58]. In the current study, organisational communication with the volunteers was inconsistent, e.g., some volunteers were not being informed about changes in their role or wider changes within the organisation. As a result, many volunteers felt unprepared for these changes and some started to ignore their commitments as communication had effectively ceased. The feeling of being isolated and disconnected affected the volunteers’ sense of belonging to the organisation. Successful communication is key to creating positive relationships between volunteers and the organisation [59] and there is evidence from the literature of the links between effective communication and nurturing commitment at various stages of the volunteer life-cycle and with any change processes implemented within an organisation [36,60]. Additionally, with regard to student volunteers in the current study, the turnover of students was perceived to be significantly higher in comparison to the volunteers who had remained in their role for some years. This contributed to dissatisfaction and a sense of isolation amongst the long-term volunteers who felt they were unable to maintain an informal network or connection with the student volunteers.

Some volunteers in the current study believed the new technology changes implemented could be more “user friendly”. One volunteer acknowledged the difficulty older volunteers may encounter with the use of new technology. Another volunteer did not mind the technological changes due to having previous experience with computers but, overall, it was suggested that the issues with technology could potentially be addressed via training sessions. Indeed, training programmes have been shown as a significant factor that helps volunteer retention [2,61] whereby the development of not only skills, but also confidence in their new role and tasks is permitted. Increasingly, technology is being used in many volunteer organisations and effective data management strategies and training needs will need to be considered [62].

As the operation of the generic adult helpline is separate from the counselling service offered to specific population groups (including people from different ethnicities and language requirements), cultural considerations were highlighted in the present study. Some volunteers felt it was necessary to have cultural understanding and support from the organisation to provide their culture-specific services. For example, there were cultural barriers such as stigma in certain callers to reach out to the mental health helpline and additional time was required to clarify what the callers’ needs were as they were sometimes framed as physical complaints, e.g., tiredness/headaches/pain instead of low mood. Additional time was also required to clarify the caller’s social situation (e.g., immigration status, integrating to a new country, social isolation or vulnerability due to dependence on their adult children). However, the generic model proposed for callers (e.g., keeping calls short or focusing only on depressive symptoms) did not consider these specific cultural considerations. Additionally, over time, these services have become isolated from the more generic helpline services due to separate communication processes.

Expectations of the difficulty of the role as well as the implications of personal issues and experiences “coming up” while on calls, had the potential to emotionally and mentally affect the volunteer. Some volunteers expected this while others were less prepared and felt there was not enough support for them in these areas. Volunteers need support systems in order to remain volunteers and continue contributing as a valuable asset to an organisation’s mission [59].

“*… I feel there is not enough support for volunteers like debriefing and receiving constructive feedback, especially for new volunteers*.”

#### 3.2.4. Intention to Stay

Volunteers’ views on positive and negative experiences were related to their role satisfaction and intention to stay. Factors including recognition, technology, communication with management/the organisation, supervision and enjoyment of the role are all considered important by the volunteers. As the factors for both job satisfaction and the intention to stay are multifactorial, the findings from the current study align with previous work whereby volunteers will remain in their role despite negative factors, provided there are positive aspects which they continued to enjoy [13].

Retention of volunteers is a significant issue for many volunteering organisations and by addressing and reducing the issues contributing to high turnover rates, organisations have the potential to enhance the consistency of the work undertaken, the organisation’s mission [63] and the overall cost-effectiveness of such organisations which often operate with limited funding. Furthermore, even highly motivated individuals can drop out of their volunteer role when the situation within or outside the organisation becomes too challenging. Whilst organisations usually advertise what volunteers can do for others, consideration of what the organisation can do for the volunteers is also important [37].

Volunteers wanted the respect and autonomy of a volunteer, not of an employee. This is consistent with the literature whereby management of volunteers is important. Volunteers expect participative leadership from their manager, allowing their feedback to be considered [64], and appreciate the idea of expressing one’s thoughts and having autonomy [13,14,41]. Not unexpectedly, managers that allow volunteers to act autonomously greatly enhance volunteers’ motivation to remain in their role [65]. As highlighted earlier, organisational communication is an important factor that affects volunteer retention and has been reported in the literature [64]. Other organisational factors are also relevant including organisational changes, change management, disconnection with volunteers, ambiguities, technological changes and recognition, acceptance and support.

“*I think that the organisation should appreciate the difference between an employee and a volunteer. The tone used to communicate with volunteers is often inappropriate, and one’s natural reaction may well be, to ignore at best or as many volunteers have done simply to decide that this is too hard and to vote with their feet*.”

The recruitment process of volunteers by an organisation and how this affects the existing volunteers’ perspectives has not been previously reported in the literature. In the current study, the recruitment of students appeared to negatively affect the long-term volunteers’ views about the connectedness of the cohort due to differences in motivation between these groups. Existing volunteers’ expectations around creating an ongoing social network was negatively affected by an obligation to volunteer [66] by student volunteers who were thought to demonstrate mainly extrinsic motivations. A significant proportion of the volunteers that were not students in the current study noticed an impact on their motivation to continue volunteering due to the high turnover of student volunteers despite holding altruistic values consistently during their time in helpline volunteering. As the students had an obligation to volunteer, prioritisation of mastery of skills such as social connectedness, self-care or self-growth may not have occurred, unlike the longer-term volunteers; this may have furthered tensions between these groups of volunteers. Additionally, some volunteers felt the organisation did not utilise the variety of skills they brought to their role. Volunteers thought they could have been placed in skill-specific roles that are consistent with their background or expertise that could have been clarified at recruitment and would be an important avenue for organisations to explore. According to the literature, while there is much focus on recruitment of new volunteers to an organisation, volunteer management and reactivation of previous volunteers also need further consideration [36].

## 4. Limitations

The final sample recruited was smaller than originally anticipated and a wider range of views could have been potentially obtained with the inclusion of more volunteers. Furthermore, the study sought helpline volunteers from only one mental health organisation, potentially leading to organisational bias. Seeking mental health helpline volunteers from other organisations would allow a greater range of perspectives to be captured, as well as allowing even greater generalisability of the findings to other volunteer organisations. Other important limitations, which are future research opportunities, is that former helpline volunteers, students or managers were not recruited. The reasons for past volunteers and students leaving their role merit further exploration and represents potential for future volunteer reactivation. While managers’ perspectives were not sought in this study, their recruitment may allow a more holistic assessment of the sector and help shape future policy. Additionally, while the service that was recruited was undergoing internal restructuring and may be viewed as a limitation, this resulted in a broader range of responses from the participants. Their responses went beyond their personal circumstances and allowed organisational factors to be explored which are relevant to other services that also rely on volunteers. This adds to the literature of organisational context affecting volunteers [67]. Additionally, organisational socialisation and social relationships have been less explored within a voluntary sector context [68] and the present findings provide further insight.

## 5. Conclusions

This study on the motivations, expectations and experiences of mental health helpline volunteers found that the majority have a high regard for their volunteer role. Factors including aspects of training, support and the enjoyment of helpline volunteering were features the volunteers appreciated within their role. Factors that have the potential to be improved are technological issues, internal communication and the recognition status of volunteers. These personal and organisational factors have the potential to impact on volunteer satisfaction and continuing in their role. These findings are potentially generalisable to other helpline volunteer organisations as well as generic volunteer organisations. Enhancing the retention of volunteers would strengthen consistency of the work undertaken, the organisation’s mission [63] and overall cost-effectiveness of such organisations which often operate with limited funding.

## Figures and Tables

**Table 1:** Focus group details.

Focus Group Number	No. of Participants	Age Range (Years)	Gender	Focus Group Facilitators	Duration (min)
1	4	44–67	4 Female	F.S., T.C., K.Z.	69
2	5	43–56	5 Female	F.S., T.C., K.Z.	60
3	5	45–56	5 Female	F.S., T.C., K.Z.	57
4	6	44–55	2 Male, 4 Female	F.S., T.C., K.Z.	53

## Appendix A

**Table A1:** Focus group and individual interview questions and sub-questions.

**1.** Introductions and explaining purpose of study
**2.** Why have you decided to volunteer? Intrinsic factors Extrinsic factors
**3.** What were your expectations when you first volunteered?
**4.** Why did you volunteer in a helpline service?
**5.** As a helplines volunteer, what are your expectations around the day to day work? hours of work weekly schedule phone calls—number of calls and content personal development supports facilities work colleagues opportunities to meet collectively to share experiences opportunities to meet with others outside work training and supervision career progression potential disappointments
**6.** Recalling your helpline volunteering experiences, what are the things you enjoyed/liked most? work environment personal development satisfaction balanced lifestyle social network
**7.** What are the things you did not like so much of your helpline volunteering position? experiences phone calls emotionally draining lifestyle balance social network volunteering schedule confidentiality
**8.** (If brought up) What are your thoughts on the organizational changes that have been occurring here? Reason for volunteer uneasiness Ways to minimize volunteer uneasiness
**9.** Do you hope to continue as a helplines volunteer? Why? aspirations and goals enjoyment life experiences societal pressure religion family values expectations met pride in organization
**10.** Other suggestions and improvements facilities timing/frequency of shifts/breaks number of phone calls rest days communication de-stress away days organizational/management issues
